# Workplace Safety Among Postgraduate Residents and Interns in India: A Cross-Sectional Assessment of Knowledge, Attitude, and Practices

**DOI:** 10.7759/cureus.98503

**Published:** 2025-12-05

**Authors:** Jamunarani Srirangaramasamy, Raghunandan Ramanathan, Arunagiri Gunasekar

**Affiliations:** 1 Department of Pathology and Laboratory Medicine, Bhaarath Medical College and Hospital, Chennai, IND; 2 Department of Psychiatry and Behavioural Medicine, Amrita Institute of Medical Sciences and Research Centre, Amrita Vishwa Vidyapeetham, Kochi, IND; 3 Department of Medical Education, Government Medical College and Hospital, Thiruvallur, Chennai, IND

**Keywords:** awareness, healthcare workers, hospital safety, medical workplace violence, occupational safety and health, workplace violence prevalence

## Abstract

Introduction

Workplace violence against healthcare professionals represents a major occupational health danger that affects workers all around the world. Medical interns and postgraduate residents face increased risks during their training period because they are new to their profession, and they work long shifts in dangerous hospital settings. The research examined knowledge, attitudes, and practices regarding workplace safety among interns and postgraduates practicing in South India.

Methods

The descriptive cross-sectional study involved 273 medical students from public and private medical schools in South India. A structured questionnaire addressed knowledge of legal protections, workplace violence exposure, psychological impact, institutional safety infrastructure, policy implementation, and support needs. Descriptive statistics, including frequencies and 95% confidence intervals, were calculated. The data analysis was conducted in IBM SPSS Statistics for Windows, Version 30 (Released 2024; IBM Corp., Armonk, New York), with a p-value < 0.05 considered significant.

Results

Among 273 participants, 47.6% reported experiencing workplace violence or harassment. The majority of incidents consisted of verbal abuse, which made up 62.3% of cases; physical violence occurred in 21.1% of incidents, and sexual harassment happened in 13.7% of cases. Only 39.2% were aware of legal protections such as the Tamil Nadu Medicare Act 2008, and 54.2% knew appropriate reporting procedures. The safety attitudes of hospital staff showed that 50.9% felt unsafe on hospital grounds, yet 76.6% experienced workplace safety-related stress and anxiety. The study found that 62.6% of respondents experienced career impact, with 40.3% rating the impact as maximum severity. The facility showed major infrastructure problems: 59.7% of respondents reported sufficient CCTV coverage, 72.9% encountered infrastructure issues, and 31.1% could access night transportation. The implementation of policies failed because only 38.8% of institutions had standard operating procedures, and 21.2% of staff members received ongoing safety training. The data show that interns face greater risks than residents across all measured categories.

Conclusions

The research identifies multiple deficiencies in legal protection knowledge, together with insufficient workplace safety measures, ineffective policy enforcement, and frequent workplace violence incidents, which affect medical students in South India. The healthcare system requires comprehensive solutions to establish safe medical training environments, as these solutions must address facility conditions, staff education, policy enforcement, and psychological support for healthcare staff.

## Introduction

Workplace violence (WPV) against healthcare workers is a significant occupational health hazard with escalating global prevalence. The World Health Organization defines WPV as "threats and assaults among healthcare workers, which include physical, sexual, verbal, and psychological abuse" [1]. Healthcare workers face disproportionate exposure to violence compared to other occupational sectors, being five times more likely to experience WPV injury than employees in other fields [2]. Healthcare workers in the United States represented 78% of all nonfatal workplace injuries from violence during 2018, which shows that healthcare facilities experience the most violent workplace incidents [1].

International surveillance data reveal that between 8% and 38% of health workers suffer physical violence at some point in their careers, with prevalence varying considerably by geographic region and healthcare setting [1]. A scoping review examining WPV across multiple healthcare contexts documented that more than 70% of hospital staff have experienced WPV in various forms, including physical assault, verbal abuse, sexual harassment, and psychological intimidation [1]. In developed healthcare systems, such as Singapore, reported WPV cases escalated from 1,080 cases in 2018 to 1,300 cases by 2020, representing a 20.4% increase [1].

WPV in healthcare manifests through four distinct types: criminal perpetrators with no legitimate relationship to the facility; client-on-worker violence (most common), where patients or family members perpetrate violence; worker-on-worker violence manifesting as bullying; and personal relationship violence extending into the workplace [2]. Studies from occupational surveillance systems document that 97% of nurses have witnessed some form of WPV, including verbal abuse, intimidation, and threatening behavior [2]. Physical assault rates vary substantially by occupational role: home healthcare workers experience 61% assault rates, nurses experience 44% assault rates, and emergency department physicians experience 21% assault rates annually [2].

Being a high-risk group, medical interns and residents constitute a particularly vulnerable subpopulation within the healthcare workforce. These trainees work extended consecutive hours, frequently exceeding 36 hours without adequate rest, engage in high-acuity clinical care in emergency and critical care settings, maintain junior hierarchical positioning within hospitals, and demonstrate limited experience in conflict de-escalation and personal safety strategies [3]. Research from South Indian tertiary care institutions demonstrates that interns experience exceptionally high burnout prevalence, with personal burnout documented in 64.05% of interns, patient-related burnout in 68.62% of interns, and work-related burnout in 40% of junior residents [3]. Medical interns experience major psychological stress at a rate of 66% because they must handle long work shifts and demanding academic requirements, heavy patient responsibilities, and weak institutional backing [3].

The psychological consequences of occupational stress and violence exposure during training years carry substantial long-term implications for professional development and career commitment. Studies examining occupational health outcomes document that healthcare worker burnout correlates significantly with reduced patient safety performance across 20 international healthcare systems over 33-year surveillance periods from 1991 to 2023 [4]. These findings suggest that negative occupational experiences during training years establish patterns of psychological vulnerability that persist throughout healthcare careers. Institutional infrastructure, safety policies, and training programs represent critical modifiable factors influencing WPV prevention. Meta-analytical evidence synthesizing 50 observational studies demonstrates that healthcare workers who received formal work safety training exhibited significantly better safety performance compared to untrained workers (odds ratio: 1.40; 95% CI: 1.01-1.95; p=0.0430) [4]. Systematic safety education programs show their ability to improve workplace safety results through this research finding.

The world faces a major problem with WPV, even though medical trainees remain vulnerable, yet South India lacks sufficient research about their knowledge and attitudes and safety practices. The previous national surveys across India have shown that healthcare workers experience high levels of violence, but researchers have not yet studied the specific dangers trainees face, nor their understanding of legal protections and their views about safety systems in healthcare facilities [5]. The research conducted a comprehensive evaluation of South Indian medical interns and residents through a cross-sectional study to assess their workplace safety knowledge, attitudes, and practices, while documenting their experiences with WPV and its psychological effects and evaluating institutional policy enforcement and trainee support requirements [5].

## Materials and methods

Study design and setting

The research involved a descriptive cross-sectional study conducted at public and private medical colleges across South India. The research aimed to evaluate medical interns' and residents' understanding and attitudes, as well as their safety practices at work. The research included various healthcare facilities across urban and rural South India because the institutions existed throughout the geographic area. The study population consisted of medical trainees working in clinical settings across various medical departments and specialties.

Sample size calculation

Sample size was calculated assuming a workplace safety knowledge, attitude, and practice proportion of 77.30% based on the study by Ahamed et al. [6]. The research employed two main elements to determine the sample size: a 5% absolute precision level and a 95% confidence level. The following formula was used as per the study by Daniel and Cross [7]: n=Z²×P×(1−P)/d², where n = sample size, Z = Z statistic for confidence level 95% is 1.960, P = expected prevalence/proportion of outcome = 0.773, and d = precision = 0.05. The required sample size was 270. The study enrolled a total of 273 medical trainees to account for potential data loss.

Study population

The study collected data from September 2025 to November 2025 among 273 medical trainees working at government and private medical colleges across urban and rural South India. Inclusion criteria were (1) medical interns or postgraduate residents, (2) actively engaged in clinical practice for a minimum of three months, and (3) willing to provide informed consent. The study excluded participants who took leave during data collection, those who declined to participate, and those who returned incomplete questionnaires. The study participants gave consent before joining the study.

Data collection

A structured self-administered questionnaire emerged from the combination of literature review, expert consultation, and pilot testing. The pilot test conducted with 30 medical trainees showed excellent results for reliability and validity testing because the total Cronbach's alpha score reached 0.795 (with subscale scores between 0.923 and 0.932), all item-total correlations exceeded 0.64, and the test-retest ICC (intraclass correlation coefficient) score reached 0.952 during the two-week interval. The questionnaire items functioned properly, so no changes were required before the main study administration.

The questionnaire was given to all eligible medical trainees and contained multiple sections that asked for the following: (1) demographic information including age and gender and professional position (intern or resident) and institution type (government or private) and location (urban or rural); (2) knowledge of workplace safety regulations including awareness of Tamil Nadu Medicare Act 2008 and similar state legislation;(3) awareness of legal protections and reporting procedures; (4) institutional practices related to workplace safety; (5) exposure to WPV including types of incidents and frequency; (6) psychological impact including stress, anxiety, and career decision influence; (7) perceived workplace safety and sense of security; (8) workplace infrastructure and safety facilities;(9) policy implementation and standard operating procedures; and (10) emergency preparedness and support needs. The researchers anonymized all responses to protect participant privacy while they maintained data integrity.

Data management and analysis

All collected data were entered and cleaned using standardized data entry protocols to ensure accuracy and consistency. The statistical analysis involved calculating descriptive statistics using frequency counts and percentage values and included 95% confidence intervals for each variable. The researchers used chi-square tests to analyze all categorical variables. The separate analyses were conducted based on gender and professional position (interns versus residents) to find out which groups faced the highest risk. The study conducted comparative analyses between government and private institutions through chi-square tests. All analyses were conducted using IBM SPSS Statistics for Windows, Version 30 (Released 2024; IBM Corp., Armonk, New York). The study used a p-value threshold of 0.05 to determine statistical significance in all analyses.

Ethical considerations

The research project commenced after receiving ethical approval from the institutional ethics committee (approval no. BEIC-213-25) and was conducted in accordance with the Declaration of Helsinki. The participants agreed to the informed consent section before joining the study, while researchers guaranteed their identity protection through anonymous participation. The study adhered to the ethical standards outlined in the Declaration of Helsinki. The research team kept participant identity hidden because they never gathered, recorded, or revealed any personal information. The research team kept all data protected by using secure storage methods that only they could access.

## Results

Study population characteristics

**Figure 1:**
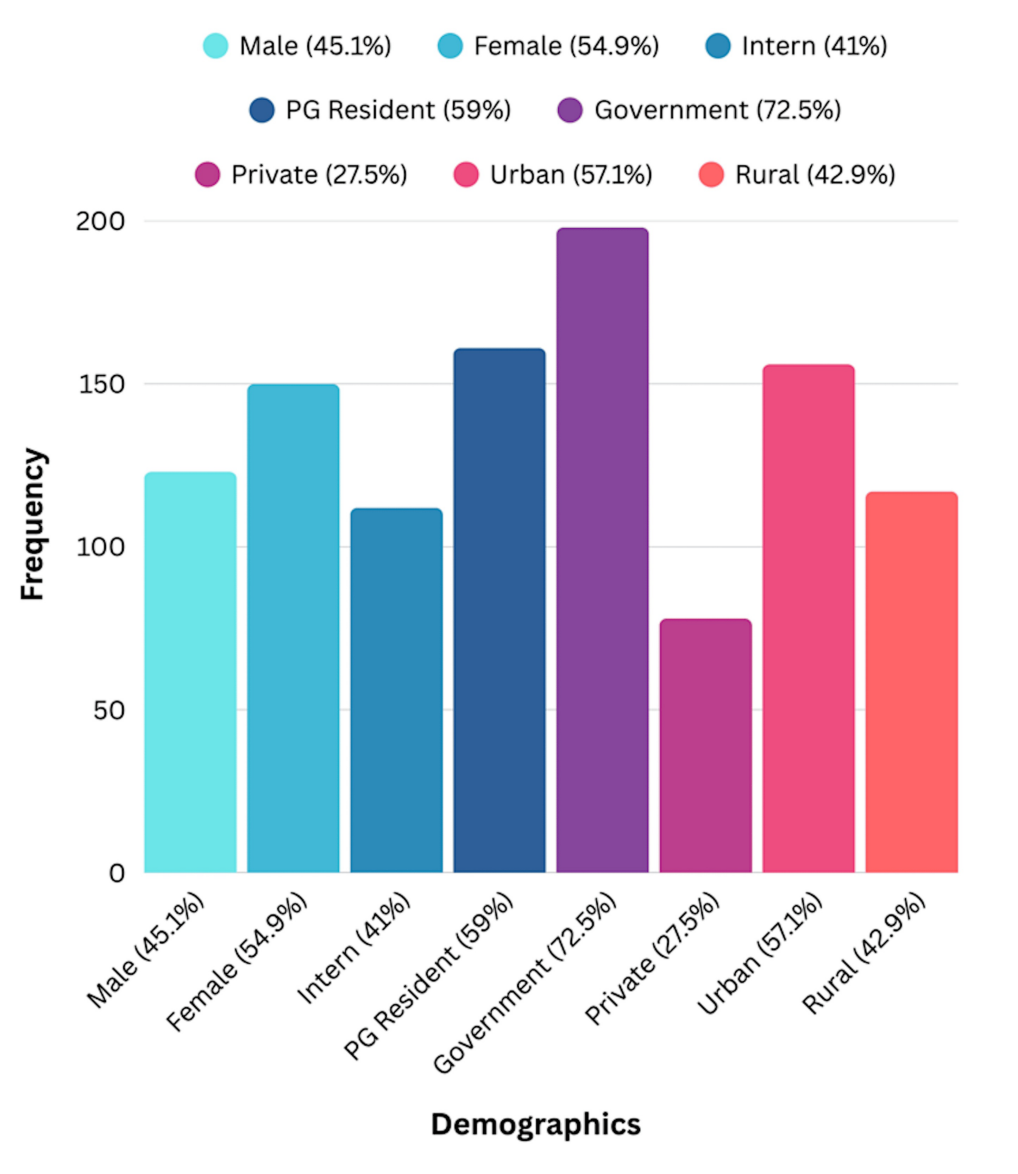
Demographic Distribution of the Study Participants

The study enrolled 273 medical trainees from public and private medical colleges across South India. The demographic distribution demonstrated significant heterogeneity: gender composition was 45.1% male (n=123) and 54.9% female (n=150); professional role included 41.0% interns (n=112) and 59.0% residents (n=161); institutional affiliation consisted of 72.5% government institutions (n=198) and 27.5% private institutions (n=75); geographic location included 57.1% urban (n=156) and 42.9% rural (n=117) settings. This distribution ensured diverse representation across multiple demographic and institutional dimensions. In Figure 1, the bar chart demonstrates the heterogeneity of the study population across multiple dimensions.

Knowledge of workplace safety regulations

Knowledge regarding workplace safety regulations and legal protections was suboptimal among the study population. Only 107 participants (39.2%; 95% CI: 33.6-45.0) were aware of the Tamil Nadu Medicare Act 2008 or similar state legislation providing legal protection against WPV. Among those aware of legal protections, perception of implementation was divided: 82 participants (30.0%; 95% CI: 24.9-35.5) perceived implementation as adequate, while 97 (35.6%; 95% CI: 30.2-41.2) perceived it as inadequate, and 94 (34.4%; 95% CI: 29.1-39.9) expressed uncertainty about implementation effectiveness. This three-way division suggests confusion regarding the actual enforcement and effectiveness of existing legal frameworks.

**Figure 2:**
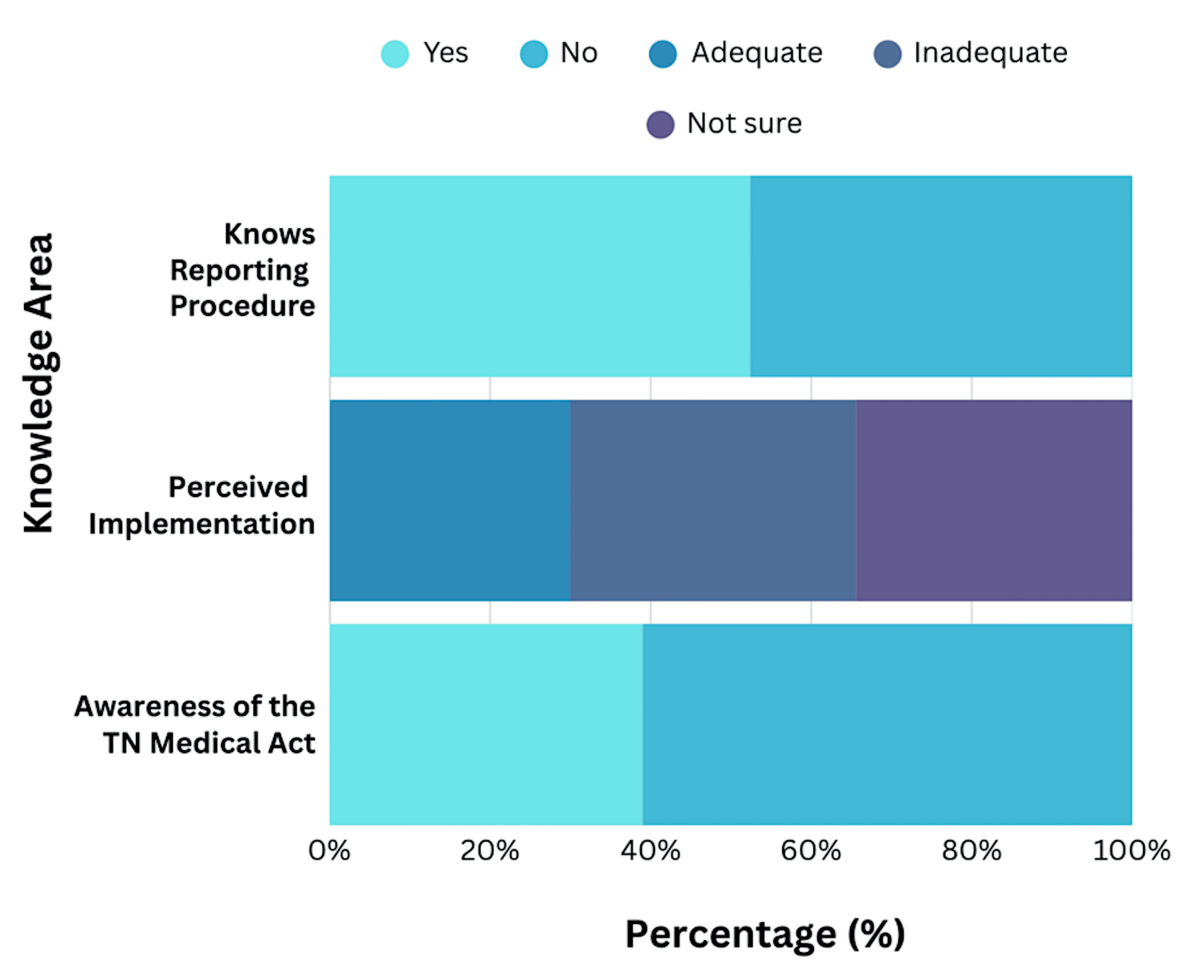
Knowledge of Workplace Safety Regulations and Reporting Procedures

Regarding awareness of reporting procedures and appropriate authorities for lodging complaints about WPV, 148 participants (54.2%; 95% CI: 48.4-59.9) reported knowing the appropriate channels, while 125 (45.8%; 95% CI: 40.1-51.6) were unaware of reporting mechanisms. This substantial knowledge gap regarding legal protections and reporting procedures suggests inadequate orientation during medical training, highlighting a critical gap in foundational workplace safety education during internship and residency programs. A detailed analysis of knowledge metrics is presented in Figure 2.

Prevalence and characteristics of WPV

A significant proportion of medical trainees reported experiences of WPV or harassment. Among 273 participants, 130 (47.6%; 95% CI: 41.9-53.3) reported being victims of WPV or harassment, while 143 (52.4%; 95% CI: 46.7-58.1) denied such experiences. This prevalence of 47.6% represents a substantial portion of the workforce exposed to violent incidents during their training years.

The data shows 254 incidents of violence against 130 victims, which means that multiple incidents affected several victims, resulting in an average of 1.95 incidents per person. The recorded violence incidents split into three main categories, which include verbal abuse as the most common form with 163 incidents representing 62.3% (95% CI: 56.8-67.5), physical violence with 55 incidents making up 21.1% (95% CI: 16.5-26.4), sexual harassment with 36 incidents at 13.7% (95% CI: 9.9-18.5), and other harassment forms with eight incidents at 3.1% (95% CI: 1.3-6.0). The most frequent form of violence in healthcare facilities worldwide manifests as verbal abuse, according to international statistics.

The 130 victims showed that 95 people (73.1%; 95% CI: 65.2-79.7) chose to take legal action after their violent experience, which reveals that most victims prefer to seek justice through official channels. The legal system created major obstacles for 38 out of 95 people, which represents 40.0% (95% CI: 30.5-50.1) who struggled with various challenges during their legal process. These barriers likely contribute to underreporting and the perpetuation of violence. Table 1 illustrates the distribution of violence types and their prevalence.

**Table 1:** Workplace Violence and Legal Proceedings (N = 273) N = total sample size (273); n = frequency; % = percentage (95%); CI = 95% confidence interval; WPV = workplace violence

Demographic Variable	Category	Frequency (n)	Percentage (%)	95% CI
Victim of Violence	Yes	130	47.6	41.9–53.3
	No	143	52.4	46.7–58.1
Violence Type	Verbal Abuse	163	62.3	56.8–67.5
	Physical Violence	55	21.1	16.5–26.4
	Sexual Harassment	36	13.7	9.9–18.5
Legal Proceedings	Yes	95	73.1	65.2–79.7
	No	35	26.9	20.3–34.8
Challenges in Process	Yes	38	40.0	30.5–50.1

Attitudes and psychological impact of workplace safety concerns

Participants' attitudes toward workplace safety revealed concerning patterns regarding both perceived safety and psychological well-being. When asked about their sense of safety on the hospital campus, responses were nearly evenly divided: 134 participants (49.1%; 95% CI: 43.3-54.9) reported feeling safe, while 139 (50.9%; 95% CI: 45.1-56.7) reported feeling unsafe. This near-50/50 distribution indicates substantial perceived vulnerability among medical trainees despite institutional safety measures.

The psychological burden associated with workplace safety concerns was pronounced. A substantial majority of 209 participants (76.6%; 95% CI: 71.5-81.2) reported experiencing stress, anxiety, or psychological distress due to workplace safety concerns, compared to only 64 (23.4%; 95% CI: 18.8-28.5) who did not. Furthermore, when asked whether workplace safety concerns influenced their career decisions, 171 participants (62.6%; 95% CI: 56.9-68.1) responded affirmatively, indicating that safety-related stress significantly impacts professional commitment and career trajectory decisions among medical trainees. Among those reporting career impact, 110 (40.3%; 95% CI: 34.8-46.1) rated this impact at the highest severity level (5/5).

This demonstrates a substantial psychological impact extending beyond immediate safety concerns to fundamental career planning and professional identity. The psychological burden is visualized in Table 2.

**Table 2:** Attitudes and Psychological Impact (N = 273) N = total sample size (273); n = frequency; % = percentage; 95% CI = 95% confidence interval

Attitude/Outcome	Response	Frequency (n)	Percentage (%)	95% CI
Perceived Safety	Feel Safe	134	49.1	43.3–54.9
	Feel Unsafe	139	50.9	45.1–56.7
Stress/Anxiety	Yes	209	76.6	71.5–81.2
	No	64	23.4	18.8–28.5
Career Impact	Yes	171	62.6	56.9–68.1
	No	102	37.4	31.9–43.1
Severity Rating	5/5 (High)	110	40.3	34.8–46.1
	4/5	61	22.3	17.7–27.6

Workplace infrastructure and safety facilities

Assessment of workplace infrastructure revealed multiple deficiencies in safety provisions. Regarding surveillance infrastructure, 163 participants (59.7%; 95% CI: 54.0-65.2) reported that closed-circuit television (CCTV) cameras were fully present in key areas (entrances, corridors), 86 (31.5%; 95% CI: 26.3-37.1) reported partial presence, and only 24 (8.8%) reported absence or were uncertain. This incomplete surveillance coverage limits the deterrent effect and investigative utility of CCTV systems.

Security personnel were reportedly present in 199 institutions (72.9%; 95% CI: 67.6-77.8), though female security guards were available in only 141 of these (51.6%; 95% CI: 45.8-57.4). The lower proportion of female security personnel is noteworthy, given the gender composition of medical trainees and the specific safety needs of female staff during high-risk situations. Regarding duty room facilities, only 121 participants (44.3%; 95% CI: 38.6-50.1) reported the availability of separate duty rooms for interns and residents, with 120 (44.0%; 95% CI: 38.3-49.8) reporting gender-specific female duty rooms. Only 114 (41.8%; 95% CI: 36.3-47.5) reported that duty rooms included attached restrooms, a basic amenity necessary for dignified and safe resting periods.

**Figure 3:**
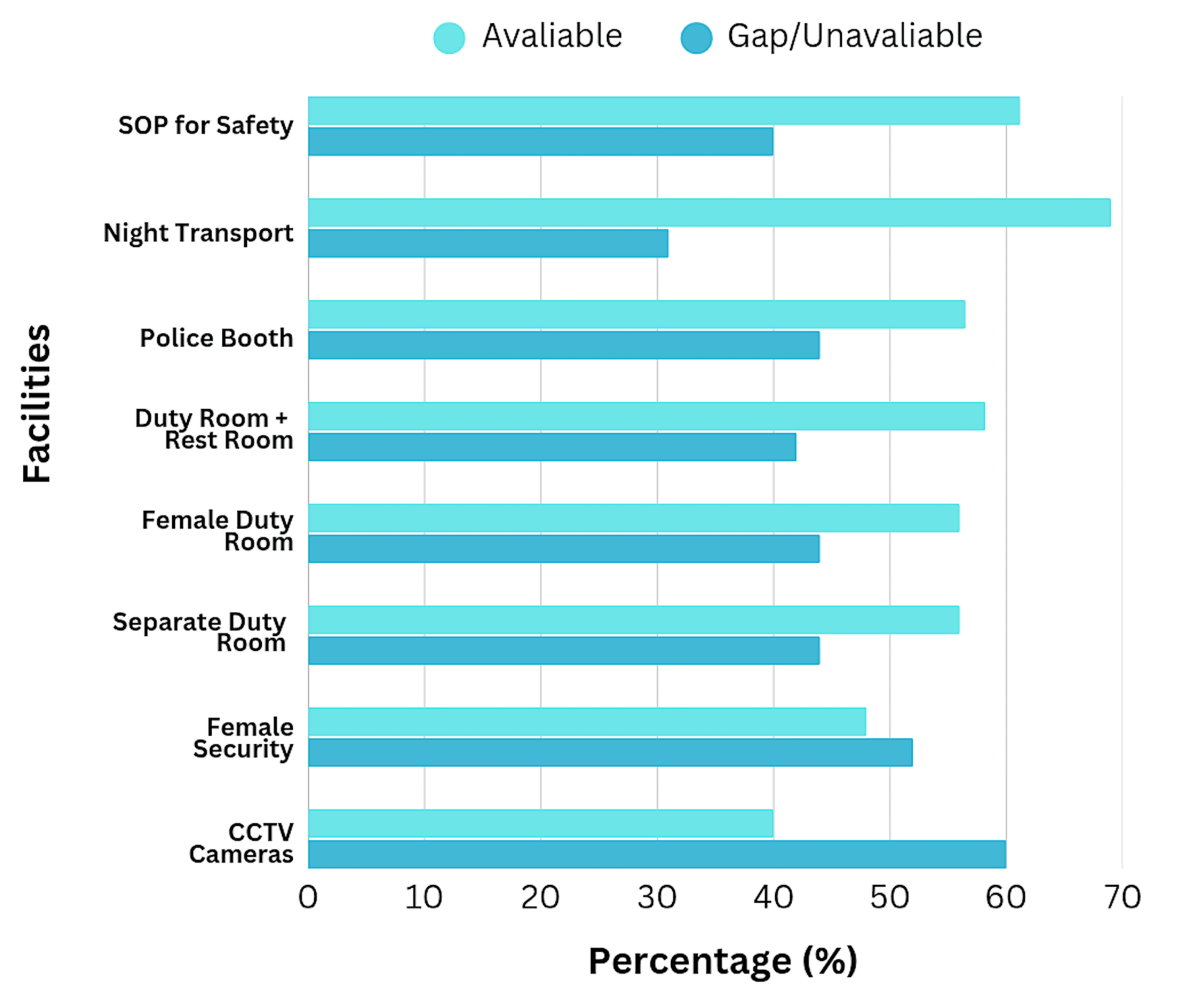
Infrastructure and Safety Facilities: Availability Versus Gaps

A substantial majority, 199 participants (72.9%; 95% CI: 67.6-77.8), reported infrastructure problems, including poor lighting, faulty locks, broken surveillance equipment, or inadequate signage. Only 78 (28.6%; 95% CI: 23.5-34.1) reported baggage screening at institutional entrances. Visitor restrictions were formally enforced in 123 institutions (45.1%; 95% CI: 39.4-50.9), while only 82 (30.0%; 95% CI: 24.9-35.5) maintained separate counselling rooms for managing agitated relatives or visitors. Most critically, only 85 participants (31.1%; 95% CI: 25.8-36.8) reported the availability of night transport facilities between hostels and hospitals, a crucial safety measure for protecting trainees during high-risk night hours. The infrastructure gaps are comprehensively illustrated in Figure 3.

Workplace safety infrastructure differed significantly between institution types, with government hospitals providing superior security and training resources compared to private facilities (Table 3). Government institutions more frequently employed security guards (76.3% vs. 64.0%, p=0.042) and provided regular safety training (24.2% vs. 13.3%, p=0.038). Both sectors demonstrated comparable deficiencies in facility-based infrastructure: separate duty rooms (≈44%), formal safety SOPs (≈37%), and female security personnel (≈50%). Neither government nor private hospitals met international standards for comprehensive workplace safety infrastructure, indicating system-wide implementation gaps requiring urgent attention across all healthcare sectors.

**Table 3:** Infrastructure by Institution Type *Asterisks denote statistical significance, p < 0.05. Comparisons between government (n=198) and private (n=75) institutions were conducted using the chi-square (χ²) test. P-values < 0.05 are considered statistically significant. Chi-square (χ²) and exact p-value are from Pearson's chi-square test of independence (df=1 for all tests). n = number of institutions; % = percentage; SOP = standard operating procedure; p = probability value

Infrastructure Element	Government (n=198)	Private (n=75)	Chi-square (χ²)	P-value
CCTV Cameras	125 (63.1%)	38 (50.7%)	3.782	0.052
Security Guards	151 (76.3%)	48 (64.0%)	4.138	0.042*
Female Guards	105 (53.0%)	36 (48.0%)	0.621	0.431
Duty Rooms	88 (44.4%)	33 (44.0%)	0.006	0.943
Duty Room + Restroom	89 (44.9%)	25 (33.3%)	3.441	0.063
SOP for Safety	82 (41.4%)	24 (32.0%)	2.338	0.127
Regular Training	48 (24.2%)	10 (13.3%)	4.323	0.038*

Policy implementation and safety training

Institutional policies addressing healthcare professional safety were inadequately implemented. Only 106 participants (38.8%; 95% CI: 33.3-44.5) reported that their institutions had formally documented standard operating procedures for healthcare worker safety. More concerning, among these 106 institutions with documented safety procedures, only 32 (30.2%; 95% CI: 21.8-39.6) conducted formal sensitization or orientation sessions on these procedures, meaning approximately 70% of institutions with written policies failed to implement proper staff education on safety protocols.

Regarding safety training, responses demonstrated a significant training delivery problem: only 58 participants (21.2%; 95% CI: 16.6-26.5) reported receiving regular or frequent safety training. The distribution showed occasional training (n=80, 29.3%; 95% CI: 24.1-34.9), rare training (n=67, 24.5%; 95% CI: 19.6-29.9), and no training (n=68, 24.9%; 95% CI: 20.1-30.3). Combined, 49.4% of participants received training rarely or never, indicating a chronic deficiency in systematic safety education. This represents a critical gap in occupational health preparedness.

Visitor management measures were inconsistently implemented: only 123 participants (45.1%; 95% CI: 39.4-50.9) reported formal restrictions on unrestricted visitor access to sensitive areas, and only 82 (30.0%; 95% CI: 24.9-35.5) reported separate counselling rooms designated for managing agitated or aggressive visitors or relatives. These findings suggest variable and inconsistent implementation of visitor management protocols across institutions. Policy implementation gaps are illustrated in Table 4.

**Table 4:** Policy Implementation and SOP Awareness (N = 273) N = total sample size (273); n = frequency; % = percentage; 95% CI = 95% confidence interval; SOP = standard operating procedure

Policy Element	Category	Frequency (n)	Percentage (%)	95% CI
Institution SOP	Yes	106	38.8	33.3–44.5
SOP Sensitization	Yes (of 106)	32	30.2	21.8–39.6
Training Frequency	Regularly	58	21.2	16.6–26.5
	Occasionally	80	29.3	24.1–34.9
	Rarely	67	24.5	19.6–29.9
	Never	68	24.9	20.1–30.3
Visitor Restrictions	Enforced	123	45.1	39.4–50.9

Comparative analysis by gender and professional role

Stratified analyses were conducted to identify particularly vulnerable subgroups. Interns (n=112) demonstrated higher vulnerability across most metrics compared to postgraduate residents (n=161). Specifically, interns reported higher victimization rates (57.1% vs. 41.0%, representing a 39% higher prevalence), greater stress or anxiety (82.1% vs. 72.7%), higher feelings of unsafety (60.7% vs. 44.1%), lower legal awareness (31.3% vs. 44.7%), lower receipt of formal standard operating procedure training (28.6% vs. 45.9%), and lower rates of regular safety training (14.3% vs. 26.1%).

Between genders, differences were generally modest but notable in some dimensions. Males reported 47.2% violence victimization versus 48.0% in females; stress or anxiety was slightly elevated in females (78.7% vs. 74.0%), and unsafe feelings were comparable (51.3% vs. 50.4%). Both groups demonstrated similar legal knowledge gaps (39.0% vs. 39.3% aware) and training receipt (20.3% vs. 22.0% regular training). However, qualitative characteristics of violence differed by gender. Government institution employees (n=198) received slightly more regular training (24.2% vs. 13.3%) and had marginally better infrastructure compared to private institution staff (n=75), though the differences were not uniformly statistically significant. Detailed subgroup comparisons demonstrating intern vulnerability are presented in Table 5.

**Table 5:** Comparative Analysis by Gender and Role N = total sample size (273); n = frequency; % = percentage; SOP = standard operating procedure

Variable	Male (n=123)	Female (n=150)	Intern (n=112)	Resident (n=161)
Experienced Violence	58 (47.2%)	72 (48.0%)	64 (57.1%)	66 (41.0%)
Stress/Anxiety	91 (74.0%)	118 (78.7%)	92 (82.1%)	117 (72.7%)
Feel Unsafe	62 (50.4%)	77 (51.3%)	68 (60.7%)	71 (44.1%)
Know Legal Protection	48 (39.0%)	59 (39.3%)	35 (31.3%)	72 (44.7%)
Formal SOP Training	46 (37.4%)	60 (40.0%)	32 (28.6%)	74 (45.9%)
Regular Training	25 (20.3%)	33 (22.0%)	16 (14.3%)	42 (26.1%)

Emergency preparedness and support needs

Assessment of emergency preparedness revealed institutional limitations. When asked whether current safety measures adequately addressed WPV prevention and response, 144 participants (52.8%; 95% CI: 47.1-58.5) agreed, while 129 (47.3%; 95% CI: 41.5-52.9) disagreed, indicating that nearly half perceived inadequate safety measures despite documented preventive infrastructure.

More concerning, only 93 participants (34.1%; 95% CI: 28.7-39.7) believed their institutions were adequately equipped to handle emergencies arising from WPV, while 180 (65.9%; 95% CI: 60.3-71.3) felt institutions were inadequately prepared. This substantial majority lacking confidence in emergency response capacity represents a critical institutional vulnerability and indicates that, despite infrastructure investments, trainees perceive inadequate emergency response readiness.

When asked about support needs, a striking 200 participants (73.3%; 95% CI: 67.9-78.2) indicated an urgent need for increased supportive staff to enhance workplace safety. Specific infrastructure improvements requested included better on-call room facilities (n=87, 31.9%; 95% CI: 26.7-37.5), enhanced security personnel (n=112, 41.0%; 95% CI: 35.3-46.9), upgraded CCTV systems (n=98, 35.9%; 95% CI: 30.5-41.6), improved lighting in common areas (n=76, 27.8%; 95% CI: 22.9-33.1), and enhanced night transportation (n=65, 23.8%; 95% CI: 19.0-29.1). These expressed needs directly correspond to documented infrastructure gaps and psychological safety concerns. Emergency preparedness gaps are visualized in Table 6.

**Table 6:** Emergency Preparedness and Support Needs (N = 273) N = total sample size (273); n = frequency; % = percentage; 95% CI = 95% confidence interval

Assessment	Response	Frequency (n)	Percentage (%)	95% CI
Current Measures Adequate	Yes	144	52.8	47.1–58.5
Equipped for Emergencies	Yes	93	34.1	28.7–39.7
Need More Support Staff	Yes	200	73.3	67.9–78.2
Need Security Enhancement	Yes	112	41.0	35.3–46.9
Need CCTV Upgrade	Yes	98	35.9	30.5–41.6

## Discussion

Prevalence and characteristics of WPV

The study found that 47.6% of medical trainees experience WPV, which matches international epidemiological data but could represent lower numbers than the actual rates among healthcare workers [8-11]. The pooled prevalence of 63% among Indian healthcare professionals reported by Hossain et al. and the documented prevalence of 62.6% in South India suggest that the lower 47.6% prevalence specifically among trainees may reflect either reduced violence targeting junior staff or, more likely, substantial underreporting due to hierarchical constraints, fear of institutional consequences, or limited awareness of reporting mechanisms [9,12]. The majority of incidents involved verbal abuse at 62.3%, which matches worldwide trends that show verbal violence as the most common type of healthcare worker abuse in various healthcare systems and locations [10,11]. The reported rate of physical violence at 21.1% stands much higher than the 8% figure found in the Indian meta-analysis by Hossain et al., yet it matches rates from other Asian healthcare settings, which indicate different levels of violence across regions [9,12]. Sexual harassment at 13.7% prevalence represents a significant occupational health concern with profound implications for affected trainees, particularly female staff members who may experience compounded vulnerability [8].

WPV shows itself as a continuous pattern of repeated trainee abuse because each victim experiences an average of 1.95 violent incidents. The psychological outcomes between repeated exposure to traumatic events and individual traumatic incidents differ because multiple traumatic events build up to create long-term emotional damage and increased sensitivity to threats and heightened alertness [8,13]. The positive outcome of 73.1% of violence victims choosing to pursue formal legal action shows that workers now better understand their rights and choose to resolve their cases through official legal processes. The systems designed to protect healthcare workers show major operational problems because 40% of legal claimants faced obstacles during their legal process, including slow investigations, institutional non-compliance, bureaucratic hold-ups, and victim intimidation [14]. The obstacles create major challenges that reduce the power of legal systems to prevent violence because they create situations where offenders can escape punishment.

Knowledge gaps in legal protections

The alarmingly low 39.2% awareness of legal protections under the Tamil Nadu Medicare Act 2008 or similar state legislation is particularly troubling, given that these protections have existed for over 15 years since the 2008 implementation [6]. The extensive knowledge gap shows that medical education programs fail to teach occupational health and legal knowledge, that institutional orientation programs focus more on paperwork than safety training, and that legislative bodies do not properly communicate protection policies to healthcare facilities [6,14]. The divided perception regarding implementation effectiveness (30.0% perceiving adequate implementation, 35.6% perceiving inadequate implementation, and 34.4% expressing uncertainty) indicates a pervasive lack of confidence in enforcement mechanisms and police responsiveness, which directly contributes to underreporting of violent incidents and perpetuation of institutional violence patterns [14].

The professional group displays inadequate knowledge of reporting procedures, as only 54.2% understand the correct reporting process, even though their legal awareness is slightly above average [6,14]. The research conducted by O'Brien et al. supports these results, as healthcare workers demonstrate poor awareness of reporting systems and encounter institutional obstacles when attempting to report incidents within their healthcare workplaces [14]. The knowledge gap reflects broader educational inadequacies, as orientation programs consisting primarily of administrative information, regulatory compliance, and procedural requirements frequently fail to adequately emphasize occupational safety education, legal protections, and conflict resolution strategies essential for trainee preparedness [14]. The international evidence shows that structured safety training programs, which combine legal frameworks with reporting procedures, personal safety strategies, and an institutional support system, lead to better awareness, higher reporting rates, and improved safety outcomes [15,16].

Psychological impact and career consequences

The research found that 76.6% of medical trainees experience psychological distress related to workplace safety, which exceeds the general occupational stress rates from previous studies with medical trainees [17,18]. Moreover, the finding that 62.6% of participants report that workplace safety concerns have influenced major career decisions, with 40.3% rating this influence at the maximum severity level, demonstrates that safety-related stress extends beyond immediate psychological symptoms to fundamentally alter career trajectories and professional commitment during critical formative years [17]. Medical training experiences create major effects on career paths, which affects both future healthcare workforce numbers and retention rates since poor training experiences drive skilled people to leave medicine or reduce their professional dedication during their early medical career stages [17,18].

The psychological effects observed in this research match with previous studies that show healthcare workers develop post-traumatic stress symptoms, burnout, clinical depression, and decreased job satisfaction after facing WPV [8,13,19]. The South Indian tertiary care institutions conducted research that revealed dangerous connections between medical interns' stress perception and their personality traits, especially when they experience WPV, because it leads to higher neuroticism and workaholism tendencies, which create harmful psychological coping mechanisms that continue throughout their healthcare careers [17]. The survey results show that 40.3% of respondents reported that their career was impacted to the highest degree, underscoring the urgent need for comprehensive psychological support systems that include counselling services, peer support networks, and occupational health resources for trainee mental healthcare [17].

Infrastructure deficiencies

The assessment discovered major infrastructure problems that produce dangerous conditions for medical trainees while threatening their safety at work. A large number of survey participants (72.9%) reported major infrastructure issues, including dim corridor and common-area lighting, defective door and window locks, non-working surveillance systems, and inadequate emergency evacuation and security signage. The ongoing infrastructure decline shows that institutional investments in infrastructure either failed to meet real operational requirements or that maintenance activities suffered from insufficient resources, disrupting organizational management and financial competition between different priorities [20]. The surveillance infrastructure assessment showed that 59.7% of participants confirmed complete CCTV camera coverage in essential areas, while 31.5% reported partial coverage and 8.8% either did not see cameras or were unsure about their presence [20]. The surveillance system gaps create major weaknesses in CCTV deterrence and reduce the system's effectiveness in investigating and prosecuting violent crimes. Modern international security standards for healthcare facilities require total CCTV monitoring of all entry points and exit points, major hallways, emergency rooms, intensive care units, and other critical clinical areas for both deterrence and forensic evidence collection [20].

The availability of separate duty rooms for interns and residents at only 44.3% of institutions falls substantially below recommended standards for healthcare workers performing extended consecutive shifts frequently exceeding 36 hours without adequate rest periods. The available duty rooms had only 41.8% of them equipped with nearby restroom facilities, which serve as fundamental resources for workers to maintain their basic hygiene needs and dignity during their long shifts. The absence of night transport facilities for 68.9% of participants creates particularly acute safety vulnerabilities, especially for female trainees who must traverse hospital grounds and nearby areas during high-risk nighttime hours. Night shift healthcare workers face their greatest safety threat from insecure transportation, according to recent occupational health surveillance studies, which specifically affect female workers who must travel through dangerous areas to reach parking lots and public transit stops [21,22].

The analysis of different institutions showed that government institutions allocate security resources at higher levels than private institutions: 76.3% of government institutions employ security guards, compared to 64.0% of private institutions (p=0.042), and 24.2% of government institutions provide regular training, compared to 13.3% of private institutions (p=0.038). The government and private sectors showed equal levels of infrastructure problems when it came to duty room availability (44.4% vs. 44.0%, p=0.943), formal safety policy documentation (41.4% vs. 32.0%, p=0.127), and female security personnel availability (53.0% vs. 48.0%, p=0.431), demonstrating that healthcare worker safety systems fail to protect workers across all institutions regardless of their funding source.

Policy implementation gaps

The critical finding that only 38.8% of institutions maintained formally documented standard operating procedures (SOPs) for healthcare worker safety represents a fundamental governance failure. The situation became more concerning because 106 institutions reported having safety SOPs, yet only 30.2% conducted formal staff training sessions to teach these procedures. The safety governance system has failed at its core because written policies without formal training programs and monitoring systems create weak protection against WPV for healthcare workers [15]. The 49.4% of trainees reporting that they receive safety training rarely or never, combined with only 21.2% reporting regular/frequent training, indicate a chronically deficient, systematic safety education that fails to adequately prepare medical trainees for the occupational hazards they will encounter [14,15].

International healthcare systems show that properly executed safety training programs lead to better safety results through increased incident reporting, decreased violence, and improved worker trust in institutional protections [15,21]. The inconsistent implementation of visitor management measures (only 45.1% reporting formal visitor restrictions in sensitive clinical areas and only 30.0% reporting dedicated counselling rooms for managing agitated or aggressive visitors) reflects highly variable institutional approaches to violence prevention, with no standardized protocols. The WPV prevention system depends on three fundamental components: visitor management systems that use identification and screening technology, designated areas for conflict de-escalation, and aggressive behavior control and emergency response systems operated by trained personnel [23,24].

Evidence-based prevention requires three integrated components: comprehensive visitor management with entry screening, dedicated de-escalation spaces with emergency communication, and trained response personnel with conflict management training [24]. Safety equipment reduces violence by 13.9 percentage points in comprehensive interventions [25].

Limitations of the study

The present study contains multiple limitations, which require full acknowledgement in this work. First, the use of a cross-sectional design limits our ability to draw causal inferences about the relationship between workplace safety factors and outcomes such as psychological stress or exposure to violence. The data represent a single snapshot in time, and longitudinal studies are required to establish temporal relationships and causality. Second, all data were collected through self-administered questionnaires, which creates a possibility for participants to provide biased self-reports. The study participants probably provided inaccurate information about their experiences because of their faulty memory and their desire to present themselves in a socially acceptable way. The presence of such bias in research can lead to inaccurate results. Third, several safety infrastructure variables depended on participant awareness and subjective perceptions, rather than direct objective assessment of the institutional environment. The method of data collection could lead to errors in measurement and produce inconsistent participant answers. Finally, although the sample size was adequate for primary analyses, some subgroup comparisons may have limited statistical power to detect subtle but important differences. Future research with larger samples and more diverse settings is recommended to confirm and extend our findings.

## Conclusions

This study reveals critical gaps in knowledge of legal protections, alarmingly high exposure to WPV, pronounced psychological burden with significant career impact, critical infrastructure deficiencies, and inadequate policy implementation regarding workplace safety among medical interns and residents in South India. The 47.6% prevalence of WPV exposure, combined with 76.6% psychological distress and 62.6% reporting career impact, demonstrates that current institutional and systemic approaches to trainee safety are inadequate.

The solution to urgent educational gaps requires a wide range of measures, including specialized teaching methods, enhanced duty room safety measures, night protection, staff training requirements, and mental health assistance programs. The correction of safety faults in public and private healthcare facilities requires immediate attention from both government institutions and private organizations, as these risks specifically affect new medical students. To ensure safe working environments with optimal medical training and high-quality healthcare delivery, it is essential to properly implement legal protections together with institutional accountability systems.

## Appendices

**Table 7:** Questionnaire of the Study

Q No.	Question	Options	Option Text
1	Email Address	-	[Open Text]
2	Consent to participate	-	I Agree
3	Age	A	< 25
		B	25-30
		C	30-35
		D	> 35
4	Sex	A	Male
		B	Female
		C	Others
		D	Prefer not to say
5	Type of Institution	A	Government Institution
		B	Private Institution
6	Role	A	CRMI
		B	Post Graduate Resident
7	City	-	[Open Text]
8	Location	A	Rural
		B	Urban
9	State	A	Tamil Nadu
		B	Kerala
		C	Karnataka
		D	Telangana
		E	Andhra Pradesh
		F	Puducherry
		G	Other [Open Text]
10	Are you aware of CCTV cameras installed in key areas of the hospital (e.g., entrances, corridors)?	A	Yes
		B	Partially Yes
		C	No
		D	Not sure
11	Are there security guards in the key areas around the campus (e.g., laboratories, emergency, ward)?	A	Yes
		B	No
		C	Not Sure
12	Are there female security guards available on duty at your workplace?	A	Yes
		B	No
		C	Not sure
	If yes, during which shifts are the female security guards typically present?	A	Day shifts only
		B	Night shifts only
		C	Both day and night shifts
		D	I am not sure
13	How effective do you find the security measures in preventing incidents of violence or harassment?	A	Very Ineffective
		B	Ineffective
		C	Neutral
		D	Effective
		E	Very Effective
14	Have you or your friend been a victim of workplace violence or harassment?	A	Yes
		B	No
	If Yes, Specify the type of violence	A	Physical
		B	Verbal
		C	Sexual
		D	Other
	If yes, have you ever been involved in legal proceedings as a result of violence encountered in your workplace?	A	Yes
		B	No
	Did you encounter any hindrances or challenges during the legal proceedings related to the violence you experienced at your workplace?	A	Yes
		B	No
15	How secure do you feel on the hospital campus?	A	Very Insecure
		B	Insecure
		C	Neutral
		D	Secure
		E	Very Secure
16	Is there a separate duty doctor room for CRMI and residents?	A	Yes
		B	No
		C	Not sure
17	Is there a separate duty doctor room for female CRMI and residents?	A	Yes
		B	No
18	Is the duty doctor's room attached to the restroom?	A	Yes
		B	No
		C	Not sure
19	Have you experienced or witnessed any safety concerns related to the infrastructure (e.g., inadequate lighting, faulty locks)?	A	Yes
		B	No
	If yes, please specify the safety concerns related to the infrastructure:	A	Inadequate lighting
		B	Faulty locks
		C	Poorly maintained facilities
		D	Isolated building
		E	Other: [Text Entry]
20	Is there baggage screening at the hospital entrance?	A	Yes
		B	No
		C	Not Sure
21	Are there designated restricted areas in your workplace where patients or their relatives are not allowed to enter, specifically for the safety and privacy of doctors?	A	Yes
		B	No
		C	Not Sure
22	Are there restrictions in place at your hospital on the number of relatives allowed to accompany a patient inside the hospital premises?	A	Yes
		B	No
		C	Not sure
23	If yes, how many persons are allowed to accompany a patient?	-	[Text Entry]
24	Does the institution have a jail duty/convict ward?	A	Yes
		B	No
	a. If yes, is the faculty accompanying you to the ward?	A	Yes
		B	No
	b. Are you feeling safe during those duties?	A	Yes
		B	No
25	Are safety protocols clearly defined and communicated?	A	Yes
		B	No
		C	Not sure
26	Do you know who to contact in case you feel threatened? (e.g., physical or emotional violence)	A	Yes
		B	No
	If yes, who will you contact? (E.g., Duty Assistant, RMO, etc.)	-	[Text Entry]
27	Is there a police booth present in your institution?	A	Yes
		B	No
		C	Not Sure
28	Is there a transport facility available for you to travel between the hospital premises and the hostel at night?	A	Yes
		B	No
		C	Not Sure
29	How satisfied are you with the current safety measures and protocols in your hospital?	A	Very Dissatisfied
		B	Dissatisfied
		C	Neutral
		D	Satisfied
		E	Very Satisfied
30	What are your maximum duty hours per shift?	A	Less than 12 hours
		B	12-24 hours
		C	24-36 hours
		D	More than 36 hours
31	How frequently do you receive updates or refresher training on safety protocols?	A	Regularly
		B	Occasionally
		C	Rarely
		D	Never
32	Is there a Standard Operating Procedure (SOP) for the safety of healthcare professionals in your institution?	A	Yes
		B	No
		C	Not Sure
	If yes, are you sensitized and oriented on the SOP for ensuring the safety of healthcare professionals?	A	Yes
		B	No
		C	Not Sure
33	Is there a separate room for patient relative counselling near the ICU to update the patient's clinical condition?	A	Yes
		B	No
		C	Not Sure
34	Do you feel there is a need to increase supportive staff to improve safety?	A	Yes
		B	No
		C	Not Sure
35	Do you think the current safety measures are adequate for handling emergencies?	A	Yes
		B	No
		C	Not Sure
36	Are you familiar with the TN Medicare Service Personnel Act of 2008 or any similar act enacted by your state?	A	Yes
		B	No
37	How effective do you think such legislation is in addressing safety issues?	A	Very Ineffective
		B	Ineffective
		C	Neutral
		D	Effective
		E	Very Effective
38	Is the implementation of the TN Medicare Service Personnel Act 2008 or similar legislation adequate in your institution?	A	Yes
		B	No
		C	Not Sure
39	Have you experienced stress or anxiety as a result of workplace safety issues?	A	Yes
		B	No
40	Do you feel that workplace safety issues influence your intention to remain in the medical profession?	A	Strongly Disagree (No influence)
		B	Disagree
		C	Neutral
		D	Agree
		E	Strongly Agree (Strong influence)
